# Sleep, Stress, Circadian Rhythm, and Diet in Pediatric Disorders of Gut–Brain Interaction: A Narrative Review

**DOI:** 10.3390/nu18172768

**Published:** 2026-08-24

**Authors:** Hubert Szyller, Maria Lasocka, Gabriela Augustynowicz, Joanna Braksator, Tomasz Pytrus

**Affiliations:** 1 2nd Clinical Department of Paediatrics, Gastroenterology and Nutrition, Wroclaw Medical University, 50-369 Wroclaw, Poland; joanna.braksator@umw.edu.pl (J.B.); tomasz.pytrus@umw.edu.pl (T.P.); 2 Student Scientific Group of Pediatric Gastroenterology and Nutrition, Wroclaw Medical University, 50-369 Wroclaw, Poland; marysialasocka@gmail.com (M.L.); gabriela.augustynowicz@interia.pl (G.A.)

**Keywords:** gastrointestinal disorders, gut–brain axis, sleep, stress, circadian rhythm, diet

## Abstract

Pediatric disorders of gut–brain interaction (DGBIs) comprise chronic or recurrent symptom-based gastrointestinal conditions that cannot be fully explained by identifiable structural, biochemical, or organic abnormalities after appropriate clinical evaluation. The term DGBI is used throughout this review in accordance with current terminology, whereas the historical term “functional gastrointestinal disorders” (FGIDs) is retained only when referring to studies that used earlier Rome classifications. This review examines the impact of four modifiable factors—sleep, psychological stress, circadian rhythm, and diet—on their development and severity in children and adolescents. Relevant literature was identified through PubMed, with emphasis on recent pediatric studies, systematic reviews, and meta-analyses. Sleep disturbances are associated with greater abdominal pain, functional disability, and impaired daytime functioning. Stress may exacerbate symptoms through hypothalamic–pituitary–adrenal axis activation, autonomic imbalance, altered motility, and visceral hypersensitivity. Circadian disruption may affect gastrointestinal motility, barrier function, enteroendocrine signaling, and microbial rhythmicity, although pediatric evidence remains limited. Diet influences fermentation, microbiota, intestinal permeability, and symptom expression, with regular meals, adequate hydration, and appropriate fiber intake representing important initial measures. These factors interact bidirectionally through shared neuroendocrine, autonomic, immune, and microbial pathways. Clinical management should therefore adopt a biopsychosocial, multidisciplinary approach combining symptom management with modification of sleep, stress, circadian habits, and dietary patterns. Further prospective pediatric studies are needed to clarify causality and support personalized interventions.

## 1. Characteristics of Functional Gastrointestinal Disorders in Children

Pediatric functional gastrointestinal disorders (FGIDs), increasingly referred to as “disorders of gut–brain interaction” (DGBIs), comprise chronic or recurrent symptom-based gastrointestinal conditions that cannot be fully explained by identifiable structural, biochemical, or organic abnormalities after appropriate clinical evaluation [1,2]. The Rome IV criteria provided an age-specific symptom-based framework that distinguished disorders of infancy and early childhood from those occurring in older children and adolescents [3,4,5,6]. In 2026, the Rome V criteria introduced an updated pediatric framework. Among the changes most relevant to this review, pediatric DGBIs are now organized predominantly according to gastrointestinal region and symptom pattern rather than the previous age-based subdivision. Rome V also revised the diagnostic criteria for several disorders discussed in this review, including irritable bowel syndrome and functional constipation, and expanded the classification of upper gastrointestinal DGBIs, including chronic nausea syndrome and functional dyspepsia [7,8]. Their pathophysiology is multifactorial and involves altered gastrointestinal motility, visceral hypersensitivity, mucosal and immune mechanisms, gut microbiota alterations, and altered central processing of visceral signals [4,9,10].

Rather than providing an exhaustive review of pediatric DGBIs, the present review focuses primarily on pain-predominant disorders in school-aged children and adolescents, particularly irritable bowel syndrome (IBS), functional dyspepsia (FD), and functional abdominal pain not otherwise specified (FAP-NOS), together with nausea-related DGBIs and functional constipation, as these are the entities most directly represented in the pediatric evidence discussed below [5,6,7,8,11]. Most studies included in this review predate Rome V and therefore use Rome III or Rome IV definitions; their original diagnostic terminology is retained where necessary to accurately describe the populations studied. Abdominal migraine remains part of the Rome IV functional abdominal pain disorder spectrum but is not a major focus of this review.

Pediatric functional gastrointestinal disorders are common, although prevalence estimates vary according to the diagnostic criteria used, participants’ age, data collection methods, and geographical region. A systematic review based on the Rome IV criteria showed that these disorders affect a substantial proportion of children, including those under 4 years of age as well as children and adolescents aged 4–18 years [11]. A meta-analysis estimated the global prevalence of functional abdominal pain disorders at 13.5%, with higher rates among girls and considerable heterogeneity between studies [12]. In a population-based study, Robin et al. reported that approximately one in four children and adolescents met the Rome IV criteria for at least one functional gastrointestinal disorder [13]. Studies comparing Rome III and Rome IV further indicate that applying Rome IV may change and in some cases reduce prevalence estimates for individual diagnoses; this should be considered when interpreting epidemiological data [14].

The clinical significance of these disorders extends beyond somatic symptoms alone. Children with DGBIs frequently experience impaired health-related quality of life, with the disease affecting physical activity, emotional functioning, family relationships, and daily participation in education [15]. School absenteeism is an important indicator of disease burden, as children with functional gastrointestinal disorders may miss school more frequently because of their symptoms, while school avoidance may further exacerbate anxiety, activity limitation, and functional disability [16]. In clinical practice, symptom overlap and the risk of symptom persistence are also important considerations; therefore, both gastrointestinal symptoms and psychosocial factors should be incorporated into diagnostic and therapeutic approaches [17]. Although functional constipation is often regarded as a benign condition, it may result in abdominal pain, abdominal bloating, fecal incontinence, toilet avoidance, family distress, and impaired quality of life in affected children [18,19].

This review focuses on sleep, psychological stress, circadian behavior, and diet because these factors are potentially modifiable and interact with gut–brain signaling through partly shared neuroendocrine, autonomic, behavioral, immune, and microbial pathways [20,21,22,23,24,25,26,27,28,29,30,31,32,33,34,35,36,37,38]. Importantly, the strength of evidence is not equivalent across these domains. Pediatric sleep data are predominantly observational [20,21,38,39,40,41,42,43], whereas psychological interventions are supported by systematic reviews and randomized controlled trials [44,45,46,47]. Dietary interventions have been evaluated in pediatric systematic reviews, although the certainty of evidence varies considerably between interventions [23,24,32,33,35]. In contrast, mechanistic evidence concerning circadian disruption and gastrointestinal function in children remains limited and is derived largely from adult or experimental studies [25,26,27,28,29,30,31]. This difference in evidential strength is reflected in Table 1.

This review summarises the current state of knowledge regarding circadian rhythm-related functional disorders, dietary factors and behavioural phenomena such as stress, thereby providing a basis for further research into a holistic approach to this group of disorders.

In this review, the literature search done between march till august 2026 focused on the relationship between sleep, psychological stress, circadian rhythm, dietary habits, and functional gastrointestinal disorders in children and adolescents. Seventy-three relevant publications were identified primarily through searches of the PubMed database using combinations of keywords related to pediatric functional gastrointestinal disorders disorders of gut–brain interaction, sleep, stress, circadian rhythm, diet, and gastrointestinal symptoms. Original research articles, randomized controlled trials, systematic reviews, and meta-analyses were considered. Particular attention was given to recent studies and publications directly addressing pediatric populations, while older or adult and experimental studies were included when they provided important mechanistic or clinical context. Additional relevant references were identified from the bibliographies of selected publications.

## 2. Sleep and Its Impact on Gastrointestinal Symptoms

Sleep is a fundamental physiological process regulating neuroendocrine, immune, metabolic, and cognitive-emotional functions; therefore, its role in pediatric functional gastrointestinal disorders should be considered within the biopsychosocial model. Sleep is a multidimensional construct, and the terms used in pediatric DGBI studies should not be treated interchangeably. In this review, “sleep duration” refers to total sleep time, sleep quality encompasses subjective satisfaction, continuity, and sleep efficiency. “Sleep timing” refers to the timing and regularity of sleep onset and awakening in relation to the circadian cycle, and “insomnia symptoms” refer primarily to difficulties initiating or maintaining sleep accompanied by impaired daytime functioning. Pediatric DGBI studies frequently assess more than one of these domains or use composite sleep-disturbance scores, which complicates comparisons between studies [38,42,43].

Schurman et al. demonstrated that clinically significant sleep problems were present in a substantial proportion of children with FGID and were associated with greater functional disability, primarily through increased physical symptom severity [20]. A study by Jansen et al. confirmed that sleep disturbances in children with FGID are associated with greater daily abdominal pain severity and increased pain-related interference with the child’s daily activities [21]. Similar findings have been reported in adolescents with functional abdominal pain, in whom coexisting sleep disturbances are associated with greater pain severity and poorer daytime functioning [39]. More recent evidence supports these observations. A systematic review of 24 studies involving 110,864 children and adolescents found that pediatric patients with DGBIs experienced more sleep problems than healthy peers and that sleep disturbances were associated with worse pain, mood, and functional outcomes [38]. Although the included studies were heterogeneous and most were of moderate or weak methodological quality, the findings support the clinical relevance of sleep assessment in pediatric DGBIs [38].

The importance of sleep is also evident across specific disorder subtypes. In a study by Kim involving children with functional abdominal pain disorders, impaired sleep quality was common and was particularly pronounced among patients with irritable bowel syndrome [40]. Colombo et al. demonstrated that, in children and adolescents with functional dyspepsia or IBS, the presence of heartburn co-occurs with sleep disturbances, anxiety, and depression. These findings suggest that the overlap of upper and lower gastrointestinal symptoms is more common among patients with a greater clinical and psychological burden [41]. A review by Friesen et al. concluded that sleep disturbances are common in children and adolescents with chronic abdominal pain; however, the quality of the available evidence is limited by heterogeneity in sleep definitions, the predominance of cross-sectional studies, and the infrequent use of objective sleep assessment methods [42]. Importantly, a 2025 scoping review of 32 studies reported sleep disturbance prevalence ranging from 19% to 75% among pediatric abdominal pain cohorts and concluded that the available evidence supports routine screening for sleep disturbances in youth with chronic abdominal pain [42]. At the same time, the authors emphasized that the extent to which sleep-directed interventions improve abdominal pain and disability remains uncertain [42].

Recent pediatric data further support the importance of distinguishing between specific sleep domains. In a school-based study of 120 adolescents, including 60 participants with FAPDs and 60 age- and sex-matched controls, youth with FAPDs reported significantly greater insomnia symptoms, general sleep disturbance, sleep-related impairment, and daytime sleepiness (*p* < 0.05 for these comparisons). Within the FAPD group, insomnia severity showed a moderate correlation with abdominal pain severity (r = 0.41, *p* < 0.01) [43]. These findings strengthen evidence for an association but remain cross-sectional and therefore do not establish the direction of causality. Importantly, recent objective data suggest that sleep abnormalities in pediatric DGBIs are not limited to subjective sleep complaints. In a 2026 case-control study using overnight polysomnography, children and adolescents with DGBIs demonstrated lower sleep efficiency after sleep onset, reduced NREM3 and REM sleep, and higher arousal and apnea-related indices than healthy controls [50]. These findings provide objective evidence of altered sleep architecture in pediatric DGBIs, although the cross-sectional design does not establish whether these abnormalities contribute to gastrointestinal symptoms, result from them, or reflect shared underlying factors.

Several interconnected biological pathways may underlie the relationship between sleep and gastrointestinal symptoms. Sleep disturbance may influence pain processing through alterations in central pain modulation, emotional regulation, autonomic activity, and hypothalamic–pituitary–adrenal (HPA) axis function. Experimental and clinical evidence suggests that insufficient or fragmented sleep may increase pain sensitivity and reduce endogenous pain inhibition, potentially increasing the perception of visceral and somatic stimuli. Evidence from pediatric chronic pain populations, a broader category that includes chronic or functional abdominal pain as well as other chronic pain conditions, indicates that insufficient or disturbed sleep is associated with greater pain intensity, functional impairment, and internalizing symptoms [51]. Within pediatric functional abdominal pain specifically, sleep disturbance has likewise been associated with greater pain severity and poorer daytime functioning [39]. These relationships appear to be bidirectional, because pain may interfere with sleep, whereas disturbed sleep may increase pain vulnerability and functional impairment on the following day [38,39,42,51].

The autonomic nervous system may represent an additional pathway linking sleep with gastrointestinal symptoms. Sleep disturbance may alter sympathetic and parasympathetic activity and increase physiological arousal, potentially affecting gastrointestinal motility and the perception of visceral stimuli. In children with functional abdominal pain and IBS, differences in autonomic regulation and sympathovagal balance have been associated with symptom severity and emotional status [52]. In children with pain-predominant FGID, associations between autonomic function and gastric motility have also been observed, further supporting the biological plausibility of interactions among sleep, stress, physiological arousal, and dyspeptic symptoms [53]. Recent evidence provides additional support for this relationship. In a 2026 study of 156 children aged 7–12 years with abdominal pain-related DGBIs, heart rate variability measures were associated with selected sleep characteristics, with some associations differing between girls and boys [54]. Lower autonomic activity was also associated with sleep-disordered breathing in both sexes [54]. These findings suggest that autonomic regulation may contribute to the sleep–DGBI relationship; however, longitudinal studies are required to determine the direction and clinical significance of these associations.

Visceral hypersensitivity represents another potential mechanism contributing to abdominal pain. Anderson et al. evaluated experimental visceral sensitivity in 101 children aged 8–15 years with medically unexplained functional abdominal pain using the water load symptom provocation task (WL-SPT) [55]. Clinical gastrointestinal symptoms, functional disability, depressive symptoms, non-gastrointestinal symptoms, and pain-related efficacy beliefs were assessed at the initial clinical evaluation, whereas visceral sensitivity was assessed approximately two months later as the increase in gastrointestinal symptoms induced by the WL-SPT. More severe baseline gastrointestinal symptoms and greater functional disability were associated with significantly greater experimentally provoked gastrointestinal symptoms (*p* < 0.04), whereas greater problem-focused pain efficacy was associated with lower laboratory visceral sensitivity (*p* < 0.01) [55]. Depressive and non-gastrointestinal symptoms were not significantly associated with laboratory visceral sensitivity. These findings support an association between clinical gastrointestinal symptom burden and experimentally evoked visceral sensitivity; however, they do not demonstrate that sleep disturbance itself causes visceral hypersensitivity. Direct pediatric evidence linking sleep disturbance to experimentally measured visceral sensitivity therefore remains limited [42,51,55].

The effects of sleep on specific symptoms, including abdominal pain, constipation, diarrhea, nausea, and appetite, are likely mediated through a combination of pain-related, autonomic, hormonal, and behavioral mechanisms. Poor sleep may exacerbate abdominal pain by lowering the pain threshold and increasing vigilance toward bodily sensations [20,21,39,51]. In patients with IBS, sleep disturbances may coexist with either constipation or diarrhea; however, pediatric studies more consistently demonstrate an association between sleep and pain severity and functional impairment than with a specific bowel habit subtype [38,40,42]. In children with IBS, available evidence does not currently support a consistent sleep-related pattern for a particular stool phenotype [40,42]. Nausea, heartburn, and dyspeptic symptoms may further impair sleep, as has been observed in children with functional dyspepsia and IBS [41]. Consequently, the relationship between sleep and gastrointestinal symptoms is likely reciprocal rather than unidirectional, with gastrointestinal symptoms potentially disrupting sleep while disturbed sleep may increase symptom burden and functional impairment [38,39,42,51].

Sleep may also interact with gastrointestinal physiology through effects on motility, appetite regulation, circadian organization, and daily behavioral patterns. Irregular sleep schedules may coincide with irregular meal timing, evening eating, and other behavioral changes that can independently influence gastrointestinal function. These factors may be particularly relevant during childhood and adolescence, when sleep schedules are influenced by school demands, extracurricular activities, evening screen exposure, and social schedules. However, the current pediatric evidence does not allow the relative contribution of these factors to DGBI symptoms to be determined. Accordingly, these pathways should be considered biologically plausible contributors rather than established causal mechanisms.

**Figure 1:**
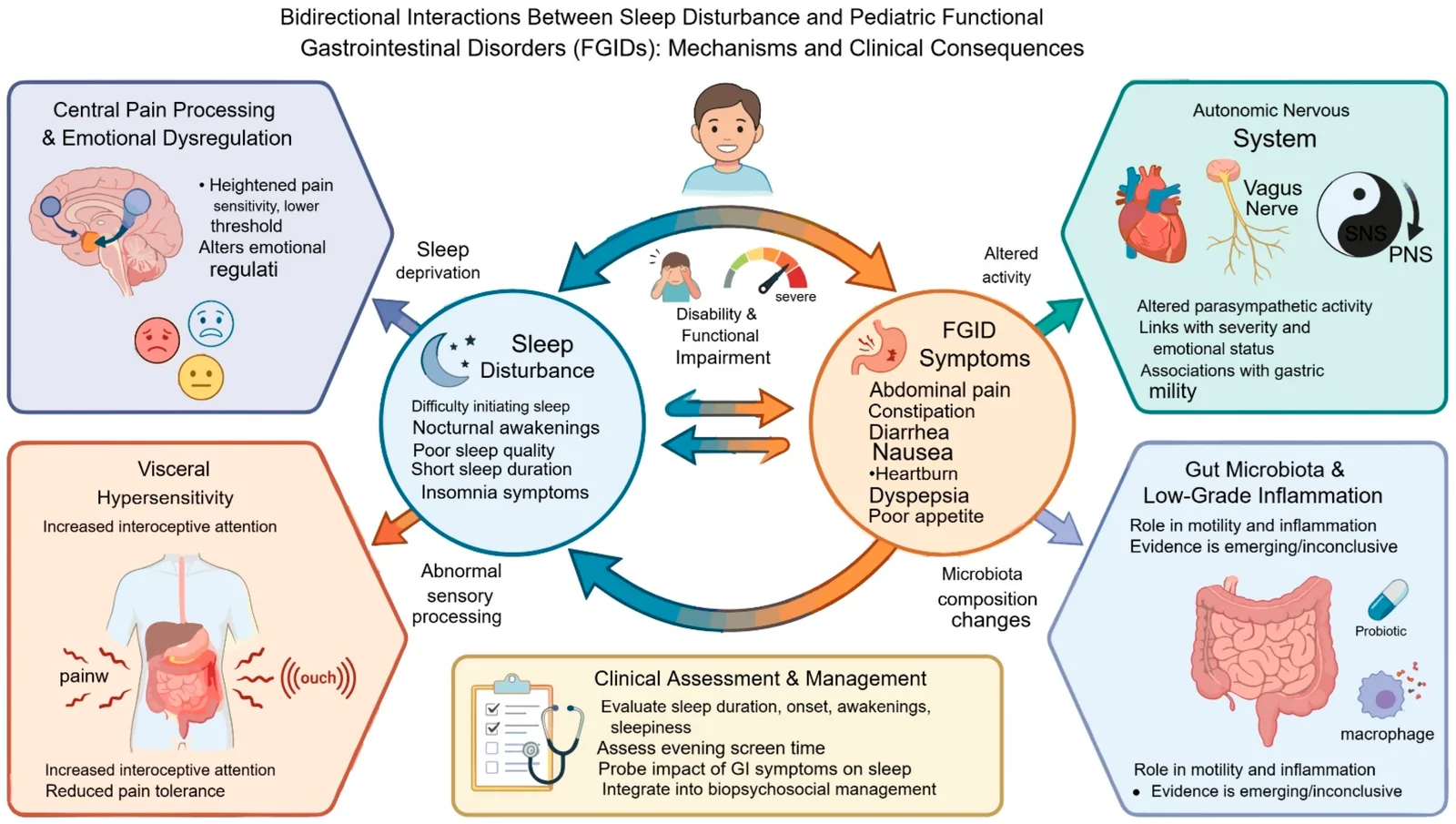
Proposed bidirectional relationships between sleep disturbances and pediatric functional gastrointestinal disorders.

These findings have practical implications for clinical assessment. The clinical evaluation of a child with an FGID should include questions regarding sleep duration, regularity of sleep onset, nocturnal awakenings, daytime sleepiness, evening screen use, and the impact of gastrointestinal symptoms on sleep [20,21,42]. Recent reviews support routine screening for sleep disturbances in children and adolescents with DGBIs or chronic abdominal pain, particularly given the high prevalence of sleep problems and their association with pain and functional impairment [38,42]. Importantly, identification of sleep disturbance does not establish it as the primary cause of gastrointestinal symptoms; rather, it may identify a potentially modifiable factor contributing to symptom persistence and impaired daily functioning. The biological mechanisms potentially linking sleep with gastrointestinal symptoms are summarized schematically in Figure 1.

Whether improvement of sleep alone reduces gastrointestinal symptoms in children remains uncertain. The available pediatric literature predominantly evaluates associations between sleep characteristics and DGBI outcomes, and adequately controlled studies designed primarily to determine whether treatment of sleep disturbance independently improves gastrointestinal symptoms remain lacking [38,42]. Therefore, sleep should currently be considered a clinically relevant and potentially modifiable treatment target within a broader biopsychosocial approach rather than an established stand-alone therapy for pediatric DGBIs. Indirect support comes from a randomized controlled trial in 60 young adults with IBS and comorbid insomnia, in which a four-week cognitive behavioral therapy for insomnia intervention improved insomnia and reduced IBS symptom severity compared with the control condition [56]. However, these findings cannot be directly extrapolated to pediatric patients. Future pediatric interventional studies should determine whether targeted treatment of sleep disturbance leads to clinically meaningful improvements in gastrointestinal symptoms, pain-related disability, and quality of life.

## 3. Stress and Its Impact on Gastrointestinal Symptoms

### 3.1. Stress Exposure and Neurobiological Stress Response

Acute and chronic stress are among the factors that may influence the course of pediatric DGBIs; however, their role should not be interpreted reductionistically as a purely ‘psychogenic’ cause of symptoms. Within the biopsychosocial model, stress is regarded as a modulator of the gut–brain axis, whereas symptom development results from interactions among biological predisposition, life experiences, emotional processes, family behaviors, the gut microbiota, gastrointestinal motility, and visceral hypersensitivity [4,22]. A review of anxiety disorders presenting gastrointestinal symptoms in children confirmed an association between anxiety and gastrointestinal symptoms while simultaneously emphasizing the difficulty in establishing a clear direction of causality [49].

Mechanistically, stress activates the hypothalamic–pituitary–adrenal (HPA) axis and the autonomic nervous system. The release of cortisol and other stress mediators may affect intestinal motility, intestinal barrier permeability, immune responses, visceral neuronal excitability, and central pain processing [22]. Although direct assessment of neuroendocrine pathways is performed less frequently in pediatric populations than in adults, the available empirical evidence supports their important pathophysiological role. This is exemplified by the findings of a randomized trial evaluating the effects of a dance-yoga intervention on salivary cortisol concentrations in girls with FAP. These findings support the use of salivary cortisol as a non-invasive research biomarker of HPA-axis activity [57]. However, salivary cortisol should not be interpreted as a validated clinical biomarker of stress severity or DGBI activity. Cortisol measurements are influenced by sampling time, collection procedures, covariate adjustment, and analytical methods, which limits comparability between studies [58]. Data regarding autonomic function in children with FAP, IBS, and pain-predominant FGID further suggest that sympathovagal balance, vagal tone, and gastric motility may play important roles in modulating pain and dyspeptic symptoms [52,53].

### 3.2. Psychiatric Comorbidity

Anxiety, depression, trauma, and somatization are particularly important considerations in the assessment of children with pain-predominant functional gastrointestinal disorders. Prospective studies by Shelby et al. demonstrated that children with functional abdominal pain are at increased risk of developing anxiety disorders during adolescence and young adulthood, even if abdominal pain resolves [59]. Walker et al. identified clinical subtypes of pediatric FAP that predicted subsequent FGID, chronic pain, and psychiatric comorbidity [60]. Longitudinal studies provide further insight into factors associated with symptom persistence. Czyzewski et al. followed 76 children aged 7–10 years with functional abdominal pain for 18–24 months and found that baseline anxiety did not predict persistence of abdominal pain frequency. In contrast, symptom-related features consistent with IBS, particularly temporal relationships between abdominal pain and stooling documented in prospective pain and stool diaries and by parental report, were associated with persistence of pain at follow-up [61]. These findings suggest that gastrointestinal symptom phenotype may be more informative for pain persistence than anxiety alone in younger school-aged children.

Extraintestinal somatic symptoms may also identify children at increased risk of persistent DGBI-related morbidity. Dengler-Crish et al. prospectively followed children with functional abdominal pain for 4–15 years. Non-gastrointestinal somatic symptoms were assessed using the Children’s Somatization Inventory and included complaints such as dizziness, back pain, headaches, and sore muscles [62]. Among 188 participants with childhood functional abdominal pain, 35.6% met Rome III criteria for an FGID at follow-up, and baseline non-GI somatic symptom burden was significantly greater among those who subsequently met these criteria [62]. Clinically, the co-occurrence of abdominal pain with multiple extraintestinal somatic symptoms may therefore indicate a broader symptom burden and identify patients who warrant closer assessment of functional impairment and longitudinal symptom persistence; however, these observational findings do not establish causality.

### 3.3. Family and School Context

School-related stress may influence gastrointestinal symptoms through academic pressure, peer conflicts, school avoidance, limited access to toilet facilities, and anticipatory anxiety related to pain or defecation. A study conducted by Pollard et al. demonstrated a seasonal relationship between functional abdominal pain disorders (FAPDs) and anxiety levels that closely paralleled the school calendar. These findings suggest that school-related stressors may substantially modulate the clinical manifestation of symptoms in a subset of pediatric patients [63]. Data on school absenteeism further indicate that school avoidance and chronic symptoms may reinforce one another: pain leads to school absence, absenteeism increases anxiety about returning to school, and disruption of daily routines perpetuates functional impairment [16,63].

Family-related stress and parental responses are also clinically relevant. Overprotective responses to pain, frequent exemption of the child from routine responsibilities, reinforcement of illness behaviors, or parental catastrophizing may exacerbate functional disability, even when motivated by genuine concern. In this context, family education should avoid portraying symptoms as merely a “psychological problem”; instead, it should explain the biologically plausible gut–brain axis model and emphasize the importance of maintaining daily routines, physical activity, adequate sleep, regular meals, and a gradual return to school [22,63].

### 3.4. Modifiable Behavioral Patterns and Psychosocial Interventions

Stress also plays a significant role in functional constipation. A systematic review by Gozali et al. demonstrated that most pediatric studies reported an association between psychological stress and functional constipation, including school-related stress, family stress, and emotional factors [48]. The underlying mechanisms may include avoidance of defecation at school, embarrassment, toilet-related anxiety, painful bowel movements, increased pelvic floor muscle tension, and reinforcement of stool-withholding behaviors [18,19,48]. In IBS and functional dyspepsia, stress may exacerbate abdominal pain, diarrhea, constipation, nausea, postprandial fullness, and heartburn through its effects on gastrointestinal motility, visceral hypersensitivity, and interoceptive attention [4,41,53,55].

**Figure 2:**
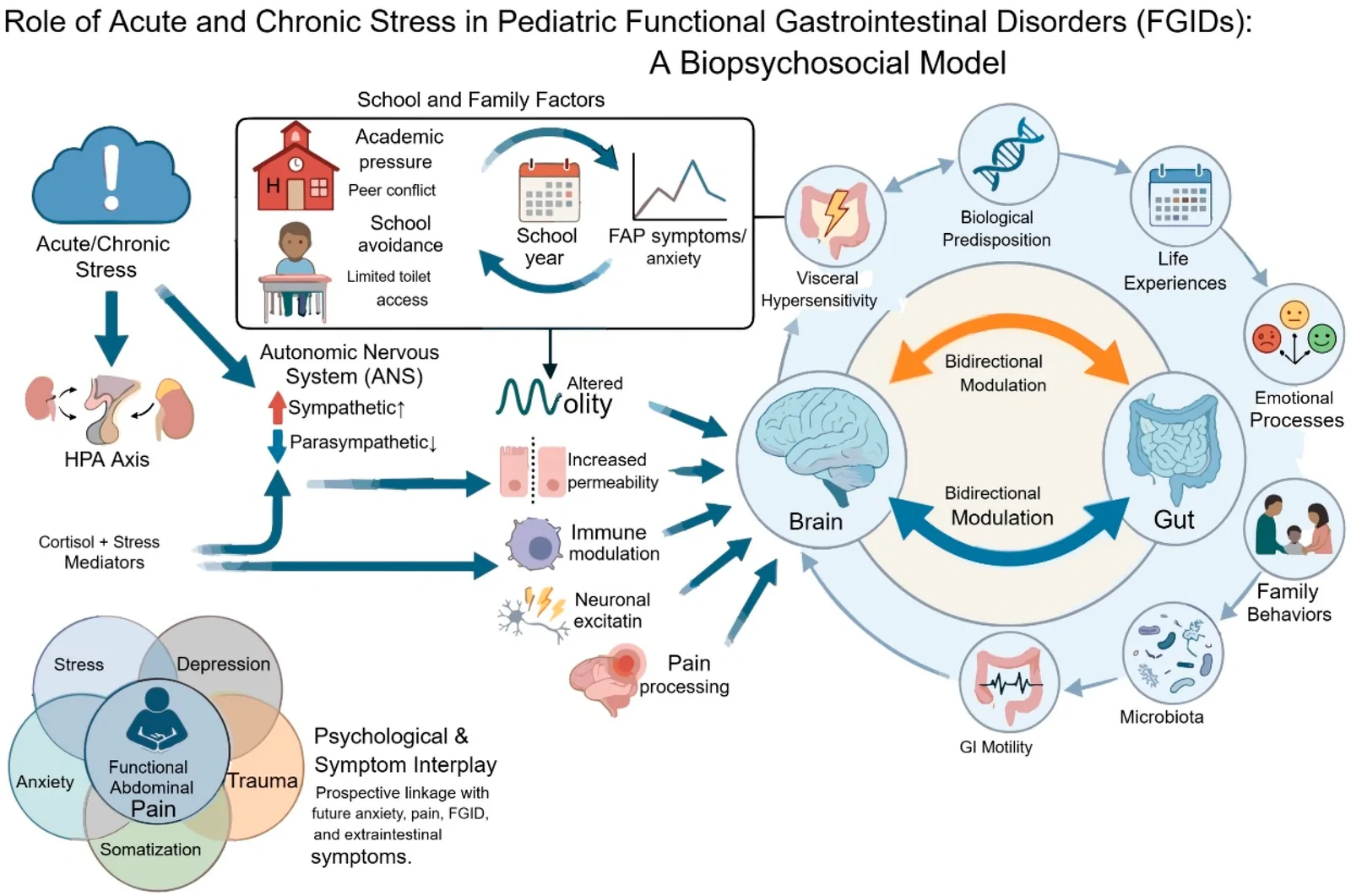
Proposed biopsychosocial mechanisms linking acute and chronic stress with pediatric functional gastrointestinal disorders.

Evaluation of the available evidence indicates varying levels of certainty. The strongest evidence relates to the prevalence of FGID, their impact on quality of life, and the effectiveness of selected psychological interventions, as these areas are supported by systematic reviews, meta-analyses, and randomized controlled trials [11,12,44,45]. A meta-analysis of psychosocial interventions for FAP demonstrated that cognitive behavioral therapy and gut-directed hypnotherapy may reduce both the frequency and intensity of pain, although the quality of evidence varies across interventions and individual studies. More specifically, CBT was associated with greater treatment success compared with no intervention (RR 2.37, 95% CI 1.30–4.34, NNT = 5, moderate-certainty evidence), whereas the estimated benefit of hypnotherapy was also favorable but supported by lower-certainty evidence (RR 2.86, 95% CI 1.19–6.83, NNT = 5) [44]. A Cochrane review of recurrent abdominal pain in children likewise supports the effectiveness of psychosocial interventions while emphasizing limitations related to small sample sizes, heterogeneous intervention protocols, and the risk of bias [45]. Randomized controlled trials evaluating gut-directed hypnotherapy and parent-involved cognitive behavioral therapy further strengthen the evidence that modulation of the stress response, parental behaviors, and coping strategies can reduce symptom severity in a subset of children [46,47]. The above is illustrated in Figure 2.

## 4. Circadian Rhythm and Chronobiology of the Gastrointestinal Tract

The gastrointestinal (GI) tract is one of the major peripheral systems regulated by the circadian system. Circadian rhythms are endogenous biological oscillations with a period of approximately 24 h that coordinate physiological processes with environmental light–dark cycles. In mammals, the central circadian clock is located in the suprachiasmatic nucleus (SCN) of the hypothalamus and is primarily entrained by light exposure, whereas peripheral clocks in the gastrointestinal tract are strongly influenced by feeding behavior, meal timing, and metabolic signals. Communication between the SCN and peripheral intestinal clocks contributes to the temporal coordination of digestion, nutrient absorption, immune responses, and host metabolism, thereby supporting gastrointestinal homeostasis [25,26,27,28]. The mechanisms underlying these processes have been demonstrated predominantly in animal models and adult studies, while direct evidence in children and adolescents remains limited. This has been summarized in Table 2 and Table 3.

At the molecular level, circadian rhythms are generated through transcriptional–translational feedback loops involving core clock genes, including CLOCK, BMAL1 (ARNTL), PER1–3, and CRY1–2. These genes regulate the rhythmic expression of downstream targets involved in epithelial renewal, intestinal hormone secretion, barrier integrity, and inflammatory responses. Intestinal epithelial cells, enteric neurons, immune cells, and enteroendocrine cells possess autonomous circadian oscillators, allowing local regulation while remaining coordinated with the central circadian system [25,26]. Evidence for these mechanisms is derived primarily from experimental and adult studies, and their specific relevance to pediatric disorders of gut–brain interaction (DGBI) remains incompletely established.

Circadian disruption caused by irregular sleep schedules, insufficient sleep duration, delayed sleep onset, night-time eating, or shift work has been associated with gastrointestinal and metabolic disturbances. Among adolescents, one common manifestation of circadian misalignment is social jetlag, defined as the discrepancy between biological and socially imposed sleep schedules, typically resulting from delayed sleep during weekends followed by earlier awakening on school days. Pediatric and adolescent research has primarily investigated associations between social jetlag, sleep disturbances, dietary behavior, obesity, and metabolic health, whereas direct evidence concerning gastrointestinal symptoms or disorders of gut–brain interaction (DGBI) remains very limited. Thus, the potential relationship between social jetlag and pediatric DGBI is currently supported mainly by indirect evidence and biological plausibility rather than by direct pediatric studies [25,29].

### 4.1. Circadian Regulation of Gastrointestinal Motility and Secretion

Virtually every component of gastrointestinal physiology exhibits some degree of circadian rhythmicity. Gastric emptying, intestinal motility, digestive enzyme secretion, bile acid metabolism, and gastrointestinal hormone release demonstrate temporal variation across the day [25,26,27,28]. Experimental and adult studies suggest that gastrointestinal motility and digestive secretions are generally enhanced during the active or feeding phase and reduced during the resting phase. Disruption of these rhythms may contribute to alterations in gastrointestinal motility and symptoms such as constipation, diarrhea, and abdominal discomfort [26,27,28]. However, direct evidence linking circadian disruption with gastrointestinal motility disorders or DGBI in children is currently limited.

Enteroendocrine hormones, including glucagon-like peptide-1 (GLP-1), glucose-dependent insulinotropic polypeptide (GIP), peptide YY (PYY), ghrelin, and serotonin, demonstrate diurnal variation and contribute to the coordination of appetite regulation, gastrointestinal motility, visceral signaling, and metabolic homeostasis with feeding cycles [25]. Experimental and adult studies suggest that alterations in enteroendocrine signaling may influence gastric emptying, intestinal transit, appetite, and visceral sensitivity, which are relevant to symptom domains commonly observed in DGBI, including abdominal pain, bloating, altered bowel habits, and meal-related gastrointestinal symptoms. In particular, altered signaling involving GLP-1, PYY, and ghrelin may affect gastrointestinal motility and appetite, while serotonin plays an important role in enteric signaling and intestinal motility [25,26]. However, these associations are supported predominantly by experimental and adult data, and direct evidence linking circadian alterations in enteroendocrine hormones to specific pediatric DGBI subtypes remains limited.

The evidence summarized in this subsection is derived predominantly from animal and adult studies. Direct pediatric evidence regarding circadian regulation of gastrointestinal motility, secretion, and enteroendocrine signaling in DGBI is scarce.

### 4.2. Circadian Rhythm and Intestinal Barrier Integrity

Maintenance of the intestinal epithelial barrier is another process influenced by circadian regulation. Tight junction proteins, epithelial regeneration, mucus secretion, antimicrobial peptide production, and intestinal immune activity display daily oscillations associated with local clock gene activity [26,29]. Experimental studies, particularly in animal models, indicate that disruption of circadian rhythms may alter the expression and organization of tight junction proteins, including occludin and claudins, and increase intestinal permeability [26,29].

Increased intestinal permeability has been proposed as one of several potential mechanisms contributing to gastrointestinal symptoms in selected functional gastrointestinal disorders; however, it should not be considered an established or universal mechanism across all pediatric DGBI subtypes. The relevance of altered barrier function may differ according to the specific disorder, symptom profile, and individual biological context. Animal studies further suggest that dysregulation of core clock genes may affect epithelial barrier function and inflammatory responses, while human evidence remains more limited and is derived predominantly from adult populations [29]. Thus, circadian effects on intestinal barrier integrity should currently be regarded as a potential mechanistic pathway rather than a general explanation for pediatric DGBI.

Evidence concerning circadian regulation of intestinal barrier integrity is derived predominantly from animal models and adult studies. Direct pediatric evidence, particularly in children with DGBI, remains insufficient.

### 4.3. Gut Microbiota and Feeding Rhythms

Accumulating evidence indicates that the gut microbiota exhibits circadian fluctuations in both microbial composition and metabolic activity. These rhythmic changes appear to be strongly influenced by feeding patterns and meal timing rather than by light exposure alone. Microbial metabolites, including short-chain fatty acids (SCFAs), may also display diurnal variation and influence intestinal immunity, epithelial metabolism, and peripheral clock gene expression [26,30]. Most evidence supporting these mechanisms comes from animal and adult studies.

Interpretation of microbiome findings in children requires particular caution because gut microbial composition and rhythmicity are influenced by several age-dependent and methodological factors. Sampling time is especially relevant because microbial abundance and metabolite production may vary across the day. In addition, recent or ongoing antibiotic exposure can substantially alter microbial composition and diversity. Age and pubertal stage are also important determinants of the developing gut microbiome, while dietary composition, meal timing, and overall nutritional patterns may independently influence microbial diversity and metabolic activity. These factors may therefore confound associations between sleep or circadian disruption and microbiome characteristics in pediatric populations.

Conversely, experimental studies indicate that irregular eating patterns, nocturnal food intake, and circadian misalignment may disrupt microbial rhythmicity and alter microbial metabolites. Such changes have been associated with metabolic dysfunction, obesity, low-grade inflammation, and gastrointestinal abnormalities [26,30]. A bidirectional relationship has also been proposed, whereby host circadian rhythms influence the microbiota while microbial metabolites and immune signaling may contribute to the synchronization of peripheral clocks. However, the clinical significance of these mechanisms in pediatric DGBI remains unclear.

Pediatric evidence is emerging but remains limited. Available studies in children and adolescents have primarily examined associations between sleep disturbances, circadian characteristics, metabolic health, and gut microbiota composition rather than gastrointestinal symptoms or DGBI. A recent systematic review of children with obstructive sleep apnea syndrome identified alterations in gut microbiota composition, supporting a possible relationship between sleep-related disturbances and the intestinal microbiome [31]. However, these findings cannot be directly interpreted as evidence that circadian disruption causes DGBI or gastrointestinal dysfunction in children. The heterogeneity of pediatric populations and potential confounding by age, pubertal development, diet, medication exposure, and sampling conditions further limits direct comparison between studies.

Evidence note: Evidence for microbiota–circadian interactions is largely derived from animal and adult studies, with emerging pediatric evidence. Direct studies investigating the relationship between circadian disruption, gut microbiota, and pediatric DGBI remain limited [26,30,31].

Compared with adult populations, evidence regarding gastrointestinal chronobiology in children and adolescents remains relatively limited. Most pediatric studies have focused on sleep duration, circadian preference, dietary behavior, obesity, metabolic health, or gut microbiota rather than direct gastrointestinal outcomes. Consequently, the potential role of circadian disruption in pediatric DGBI is currently supported mainly by biological plausibility and extrapolation from adult and experimental research.

Studies specifically evaluating social jetlag and gastrointestinal physiology in children are scarce, and direct evidence linking circadian disruption with intestinal permeability, gastrointestinal motility, or DGBI in pediatric populations remains insufficient. Therefore, regular sleep–wake schedules and consistent meal timing may represent plausible strategies for supporting gastrointestinal and metabolic health, but evidence demonstrating that these measures prevent or improve pediatric DGBI is currently lacking. Further longitudinal and interventional studies in pediatric populations are required to determine whether circadian regulation represents a clinically relevant modifiable factor in the development and management of DGBI [25,26,27,28,29,30,31].

## 5. Diet and Eating Habits

Diet is one of the important modifiable factors that may influence symptoms of disorders of gut–brain interaction (DGBIs). Beyond providing nutrients, dietary patterns may affect gastrointestinal motility, microbial composition, fermentation processes, intestinal permeability, and gut–brain signaling, potentially contributing to abdominal pain, bloating, and altered bowel habits [32,33,34]. However, evidence derived from general metabolic, microbiome, or adult gastrointestinal research should be distinguished from direct evidence in children with DGBIs.

Western dietary patterns characterized by a high intake of ultra-processed foods, refined carbohydrates, saturated fats, and sugar-sweetened beverages have been associated with poorer gastrointestinal and metabolic health and with alterations in gut microbial composition. In contrast, dietary patterns rich in fruits, vegetables, legumes, and whole grains, including Mediterranean-style diets, may support overall metabolic and gastrointestinal health through increased intake of fiber and bioactive compounds and potential effects on the gut microbiome [33]. However, direct evidence demonstrating that specific dietary patterns prevent or improve pediatric DGBIs remains limited, and associations observed in adult or general-population studies should not be interpreted as established causal relationships in children.

Meal timing may also influence gastrointestinal function. Irregular eating patterns, prolonged fasting, and breakfast skipping may affect gastrointestinal motility, circadian regulation of digestive processes, and overall dietary quality. Although direct pediatric evidence linking meal timing with DGBI symptoms is limited, regular meal consumption is commonly included in the dietary management of pediatric DGBIs [33]. Thus, regular meal patterns may represent a reasonable low-risk component of symptom management, while the magnitude of their specific therapeutic effect in children remains uncertain.

Dietary fiber plays an important role in maintaining gastrointestinal health, but its effects depend on the amount, type, and individual tolerance. Adequate dietary fiber intake as part of a balanced, age-appropriate diet is generally recommended in children. This should be distinguished from therapeutic fiber supplementation, which may be considered in selected patients with specific symptoms or disorders. Soluble, gel-forming fibers such as psyllium may improve stool consistency and bowel function and have been studied in functional constipation and functional abdominal pain disorders [23,32,33]. In contrast, some poorly tolerated or excessive amounts of insoluble or highly fermentable fiber may increase gas production, bloating, or abdominal discomfort in susceptible individuals. Therefore, fiber intake should be individualized according to age, baseline dietary intake, predominant symptoms, and tolerance rather than increased indiscriminately.

Among dietary interventions, the low-fermentable oligosaccharides, disaccharides, monosaccharides, and polyols (low-FODMAP) diet has received considerable attention. FODMAPs are poorly absorbed carbohydrates that increase luminal water content and undergo bacterial fermentation, potentially leading to gas production, intestinal distension, and gastrointestinal symptoms. Although the low-FODMAP diet has demonstrated efficacy in adults with irritable bowel syndrome, evidence in children remains limited and heterogeneous. Available pediatric trials include relatively small sample sizes and provide low-certainty evidence [24,32,33,35]. Therefore, routine implementation of a strict low-FODMAP diet is not recommended in children. In selected patients with persistent symptoms, it may be considered under the supervision of an experienced pediatric dietitian, with an appropriately structured elimination and reintroduction phase to minimize nutritional risks [32,33,35].

Before initiating restrictive or elimination diets, an appropriate clinical assessment should be performed to exclude relevant organic diseases when indicated by the clinical presentation. This may include evaluation for conditions such as celiac disease, particularly when symptoms, growth abnormalities, anemia, family history, or other clinical features raise suspicion. Elimination of gluten should therefore not be initiated before appropriate assessment for celiac disease when clinically indicated, as dietary gluten restriction may interfere with subsequent diagnostic testing.

It is also important to distinguish food intolerance or hypersensitivity from food allergy. In children with DGBIs, symptoms attributed to specific foods may be related to carbohydrate malabsorption, intestinal fermentation, altered gastrointestinal motility, or visceral hypersensitivity rather than IgE-mediated food allergy [32,33]. Consequently, routine elimination of gluten, lactose, or other foods without an appropriate clinical indication is not supported by current evidence.

Restrictive diets require particular caution in pediatric populations because they may lead to inadequate intake of energy, calcium, vitamin D, iron, zinc, and dietary fiber and may increase the risk of disordered eating and impaired growth. Therefore, current approaches emphasize individualized dietary counseling rather than empiric food exclusion. Initial management should focus on establishing regular meal patterns, maintaining adequate hydration, ensuring age-appropriate dietary fiber intake, and reducing excessive consumption of ultra-processed foods and sugar-sweetened beverages before considering more restrictive dietary interventions [32,33,35]. The above has been summarized in Table 4.

## 6. Reciprocal Interactions Between Sleep, Stress, Circadian Rhythm, and Diet: An Integrative Model

Sleep, stress, circadian rhythm, and diet are closely interconnected components of the gut–brain axis. Rather than acting as isolated factors, they may influence gastrointestinal function through overlapping neuroendocrine, autonomic, immune, metabolic, and microbial pathways, thereby contributing to the persistence and severity of disorders of gut–brain interaction (DGBIs) [34,36,37]. The main interactions are summarized in Table 2.

Among these factors, the relationship between psychological stress and sleep is relatively well established. Psychological stress activates the hypothalamic–pituitary–adrenal (HPA) axis and the sympathetic nervous system, while chronic stress may impair sleep quality and promote sleep fragmentation. Conversely, insufficient or disrupted sleep can increase stress reactivity and HPA-axis activity, potentially creating a reinforcing cycle [34,36]. These alterations may affect gastrointestinal motility, visceral sensitivity, intestinal immune signaling, and barrier function, which may contribute to gastrointestinal symptoms in susceptible individuals [34,36].

Circadian timing provides an additional layer of interaction. Irregular sleep–wake schedules and social jetlag may be accompanied by delayed or irregular meal timing, breakfast skipping, and less favorable dietary choices [25,26,27,28]. Conversely, late or irregular food intake may influence peripheral circadian rhythms in the gastrointestinal tract and metabolic tissues. Although these relationships are biologically plausible and supported by experimental and adult data, the extent to which circadian misalignment directly contributes to pediatric DGBIs remains insufficiently established [25,26,27,28,37].

Diet and sleep may likewise influence each other. Dietary composition and meal timing have been associated with sleep duration and quality, while inadequate sleep may alter appetite regulation and food preferences, potentially favoring energy-dense foods [37]. Dietary patterns characterized by greater intake of fiber and plant-based foods may additionally support microbial metabolism and short-chain fatty acid (SCFA) production, whereas highly processed dietary patterns may have opposite effects. However, the clinical relevance of these pathways for individual pediatric DGBI subtypes remains incompletely defined [37].

The gut microbiota may represent one of the pathways through which these factors converge. Sleep disruption, psychological stress, circadian misalignment, and dietary changes have each been associated with alterations in microbial composition or function, while microbiota-derived metabolites may participate in immune, metabolic, and gut–brain signaling [36,37,38,64,65,66,67]. Nevertheless, in children with DGBIs, the direction and clinical significance of these associations remain uncertain. Pediatric studies have demonstrated differences in the microbiota of patients with functional constipation, IBS, and FAPDs, but the findings are heterogeneous and are influenced by factors such as age, pubertal stage, diet, medication and antibiotic exposure, and sampling conditions [64,65,66,67]. Therefore, a direct causal pathway linking lifestyle-related disruption to microbiome changes and subsequently to DGBI symptoms should not yet be assumed.

Taken together, the available evidence supports a reciprocal model in which sleep, psychological stress, circadian timing, and dietary behavior may reinforce one another and interact with gut–brain signaling. However, the strength of evidence differs across individual pathways. The bidirectional relationships between stress and sleep and the influence of diet and meal timing on circadian physiology are relatively well supported, whereas microbiome-mediated pathways and their specific contribution to pediatric DGBIs remain largely mechanistic or indirect. Consequently, clinical management may benefit from considering these factors together rather than in isolation, although further pediatric intervention studies are needed to determine whether multicomponent lifestyle interventions provide greater benefits than single-component approaches [25,38]. The main model is presented in Table 5.

## 7. Clinical Implications and Current Limitations

The management of pediatric disorders of gut–brain interaction (DGBIs) should adopt a biopsychosocial approach, integrating gastrointestinal symptoms with lifestyle, psychological, developmental, and environmental factors. The clinical evaluation of a child with suspected DGBI should proceed in a stepwise and age-appropriate manner rather than being framed as a choice between no investigation and an extensive diagnostic work-up. Before assigning a positive DGBI diagnosis, clinicians should characterize symptom onset, duration and severity, growth trajectory, stool and vomiting patterns, relationships of symptoms to eating and defecation, dietary and medication exposures, psychosocial context and relevant family history, followed by a focused physical examination [5,17,68,69,70]. The threshold, urgency, and extent of further investigations depend on the child’s age, clinical phenotype, symptom severity, growth, and the presence of alarm features.

Alarm features that should prompt consideration of targeted investigation include involuntary weight loss or deceleration of growth, delayed puberty, gastrointestinal bleeding, excessive or persistent vomiting, severe chronic diarrhoea, persistent right upper- or right lower-quadrant pain, perianal disease, dysphagia, unexplained fever or arthritis and a family history of inflammatory bowel disease or celiac disease [5,17,68,69,70]. Depending on the clinical presentation, targeted investigations may include complete blood count, inflammatory markers, faecal calprotectin or lactoferrin, celiac serology, liver or pancreatic tests, urinalysis, imaging, or endoscopy [70]. In the absence of alarm features and when the symptom pattern is compatible with current DGBI diagnostic criteria, routine extensive testing is generally of low yield; however, limited investigations may still be appropriate depending on age, symptom phenotype, and diagnostic uncertainty [17,68,69,70].

First-line management should emphasize non-pharmacological interventions. Families should be encouraged to establish regular sleep schedules, maintain consistent meal timing, consume a balanced diet with adequate fiber and fluid intake, increase daily physical activity, and limit evening caffeine consumption and screen exposure. These low-risk interventions may improve gastrointestinal symptoms while also benefiting mental and metabolic health [17,18,23,32,33,34,35,38,44,45,46,47,69].

Physical activity and screen exposure should be considered secondary supportive lifestyle domains in the present review because direct evidence linking them independently to pediatric DGBI outcomes is substantially more limited than the evidence available for sleep, stress, and dietary factors. In a small cross-sectional study of adolescents with functional abdominal pain, total daily screen time did not correlate with the severity of abdominal pain or other gastrointestinal symptoms [71]. Conversely, an accelerometer-based study of preschool children suggested an association between higher moderate-to-vigorous physical activity and more favorable defecation habits [72]. However, this study did not evaluate Rome-defined DGBIs and cannot establish causality. Consequently, recommendations regarding physical activity and screen exposure should primarily be framed as components of general health, maintenance of daily routines, and sleep hygiene rather than as established DGBI-specific treatments.

Referral to additional specialists should be considered when symptoms persist despite initial management or when specific problems are identified. Children with suspected nutritional deficiencies, restrictive eating patterns, or those requiring dietary interventions such as a low-FODMAP diet should be referred to a registered pediatric dietitian. Patients with significant anxiety, depression, chronic stress, or maladaptive coping strategies may benefit from evaluation by a psychologist, particularly for cognitive behavioral therapy or gut-directed hypnotherapy. Referral to a pediatric gastroenterologist is indicated in the presence of alarm features, diagnostic uncertainty, treatment failure, or suspected organic gastrointestinal disease [17,18,32,33,34,35,44,45,46,47,69].

Overall, current evidence supports a multidisciplinary, patient-centered approach that targets modifiable lifestyle factors alongside symptom management rather than focusing exclusively on gastrointestinal manifestations.

### Limitations of Current Evidence

Despite increasing recognition of the role of lifestyle factors, including diet, sleep, stress, and circadian regulation, in pediatric disorders of gut–brain interaction (DGBIs), several limitations affect the interpretation of current evidence. Most available studies are observational, making it difficult to establish causal relationships between lifestyle exposures and gastrointestinal symptoms. Associations between poor sleep, psychological stress, dietary patterns, and DGBIs may reflect bidirectional interactions, where symptoms themselves contribute to sleep disruption, altered eating behavior, and increased psychological burden [32,33,38,42,44,45,69].

Another important limitation is the heterogeneity of diagnostic criteria and assessment methods. Studies have used different definitions of functional gastrointestinal disorders, including Rome III and Rome IV criteria, which complicate comparisons between populations. Similarly, evaluation of sleep quality, stress levels, dietary intake, and physical activity is frequently based on self-reported questionnaires, parental reports, or heterogeneous measurement tools, limiting the accuracy and reproducibility of findings [11,14,32,33,38,42,68,69].

Additional sources of heterogeneity and confounding should also be considered. Pediatric cohorts often span broad age ranges, and while developmental and pubertal stage may influence sleep patterns, circadian timing, psychological responses, and symptom reporting, these variables are not consistently stratified or adjusted for across studies [11,38,42,43]. Psychiatric comorbidity represents another major potential confounder because anxiety, depression, and somatic symptoms are associated with both DGBI severity and sleep- or stress-related outcomes [38,49,59,60,61,62]. Consequently, associations attributed to an individual lifestyle factor may partly reflect shared psychological or developmental determinants.

Microbiome-related studies are additionally vulnerable to confounding by antibiotic and probiotic exposure. A recent systematic review of 89 studies involving 9712 children found that systemic antibiotic exposure was consistently associated with alterations in gut microbial composition and reduced alpha diversity, with some effects persisting for months after treatment [73]. Probiotic interventions are likewise highly heterogeneous with respect to strain, dose, treatment duration, and outcome assessment [67]. Failure to adequately record or adjust for these exposures may therefore contribute to inconsistent microbiome findings across pediatric DGBI studies.

Socioeconomic and cultural context may further limit generalizability. Earlier pediatric meta-analytic data demonstrated substantial geographic variability and inconsistent associations between socioeconomic status and functional abdominal pain [12]. More recent global evidence confirms marked between-study heterogeneity: a meta-analysis of 67 studies involving 1,712,737 children and adolescents from 34 countries estimated an overall FAPD prevalence of 10.89% (95% CI 9.51–12.48%), with very high heterogeneity (*I*^2^ = 95.90%) [74]. Such variation may reflect differences in diagnostic criteria, sampling, symptom reporting, healthcare access, socioeconomic conditions, and cultural context and should caution against assuming uniform associations across populations. Publication bias and selective-reporting bias should also be considered, particularly in areas dominated by small observational studies or intervention trials [32,33,38,44,45,75].

A major challenge remains the limited availability of high-quality pediatric studies. Compared with adults, children and adolescents are underrepresented in clinical trials investigating dietary interventions, microbiota-targeted therapies, circadian disruption, and psychological approaches. Many mechanistic insights regarding gut microbiota, intestinal permeability, and neuroendocrine pathways are derived from adult populations or experimental models, and their direct applicability to pediatric patients remains uncertain [32,33,38,42,44,45,67,69].

Furthermore, determining the directionality of associations remains difficult. For example, it is unclear whether sleep disturbances and dietary irregularities contribute primarily to the development of gastrointestinal symptoms or whether chronic abdominal pain and altered bowel function lead to secondary changes in sleep, stress, and eating behaviors. Longitudinal studies are needed to clarify causal pathways and identify critical periods during childhood when lifestyle interventions may have the greatest preventive impact [32,33,38,42,69].

Finally, sleep, psychological stress, diet, and circadian behavior should not be considered independent exposures. Sleep timing affects meal timing and circadian alignment. Stress may affect both sleep and dietary behaviors, while the timing of food intake may, in turn, influence peripheral circadian rhythms and sleep [34,36,37]. Consequently, conventional observational analyses may have difficulty separating direct effects from confounding, mediation, and interaction among these variables. Future prospective studies should therefore use repeated and, where possible, objective measurements of several lifestyle domains simultaneously and apply analytical approaches capable of evaluating their independent and combined effects.

Future research should focus on large-scale prospective pediatric cohorts using standardized diagnostic criteria, objective measures of sleep and circadian rhythms, validated dietary assessment tools, and integrated analysis of microbiome, immune, metabolic, and psychological parameters. Such studies are essential to develop personalized, evidence-based interventions targeting the gut–brain axis in children with DGBIs.

## 8. Conclusions

Pediatric disorders of gut–brain interaction (DGBIs) are common, clinically relevant conditions diagnosed primarily using symptom-based Rome criteria. Although the terminology and classification have evolved with Rome V, much of the pediatric evidence remains based on Rome III or Rome IV definitions. Across the available literature, sleep disturbance, psychological stress, and symptom-related functional impairment have the most consistent pediatric support, with associations involving abdominal pain, disability, quality of life, and school functioning. These relationships are bidirectional, as gastrointestinal symptoms may also disrupt sleep, daily functioning, and emotional well-being.

Several other mechanisms, including circadian misalignment, altered intestinal permeability, and microbiome-mediated gut–brain signaling, are biologically plausible but less firmly established in pediatric DGBIs. In particular, evidence linking social jetlag, meal timing, or sleep-related circadian disruption directly to specific pediatric DGBI outcomes remains limited, and much of the mechanistic evidence is derived from adult or experimental studies. Similarly, microbiome findings in children are heterogeneous and do not yet establish a consistent causal pathway.

From a clinical perspective, assessment should begin with an age-appropriate history and physical examination, including evaluation for alarm features. In children without alarm features whose symptoms are consistent with a DGBI, extensive routine investigations are generally not required, although targeted testing should be guided by the clinical phenotype and diagnostic uncertainty. Management should emphasize education, regular sleep and meal schedules, adequate hydration and fiber intake, physical activity, and individualized psychological or dietary support. Restrictive diets, including low-FODMAP approaches, should be reserved for selected patients and implemented with appropriate pediatric dietetic supervision.

Key research priorities include prospective pediatric studies using standardized diagnostic criteria and objective measures of sleep, circadian timing, and dietary intake, together with longitudinal assessment of microbiome, immune, metabolic, and psychological factors. Such studies are needed to clarify causality, identify clinically relevant subgroups, and determine whether targeting multiple modifiable lifestyle factors improves outcomes in pediatric DGBIs.

## Figures and Tables

**Table 1:** Selected pediatric DGBIs, modifiable factors, and strength of supporting evidence.

Clinical Entity	Potentially Relevant Modifiable Factors	Type of Evidence	Representative Quantitative Findings	Interpretation of Evidence
Functional abdominal pain/FAPD	Sleep quality/insomnia, pain coping, school-related stress, anxiety, family responses	Pediatric observational studies, systematic reviews, RCTs/meta-analysis for psychological interventions [20,21,38,39,43,44,45,46,47]	In [21], 61% exceeded the clinical cutoff for sleep disturbance, children above the cutoff had greater pain severity (3.5 ± 1.2 vs. 3.1 ± 0.9, *p* = 0.043) and pain interference (0.7 ± 0.4 vs. 0.4 ± 0.3, *p* = 0.003). In [43], insomnia severity correlated with abdominal pain (r = 0.41, *p* < 0.01). CBT increased treatment success versus no intervention: RR 2.37 (95% CI 1.30–4.34), NNT = 5 [44].	Moderate evidence for association, stronger evidence for selected psychological interventions; causality of sleep associations remains uncertain
Irritable bowel syndrome	Sleep quality/insomnia, stress regulation, bowel habits, dietary triggers	Mainly observational sleep studies, systematic reviews of sleep and diet [32,33,38,40]	In [40], clinically relevant poor sleep occurred in 71.4% of patients with IBS versus 47.4% with FAP-NOS. Across the systematic review [38], most IBS-specific sleep studies were of weak or moderate methodological quality.	Low-to-moderate evidence for sleep associations; dietary intervention evidence heterogeneous
Functional dyspepsia	Meal patterns, individual food tolerance, anxiety, sleep disturbance	Cross-sectional observational evidence, dietary systematic reviews [32,33,41]	In children with FD and/or IBS, heartburn was associated with greater difficulty initiating/maintaining sleep (17.05 ± 5.68 vs. 15.04 ± 4.89, *p* = 0.007, Cohen’s d = 0.379) [41].	Mainly observational, effect sizes small-to-moderate and affected by symptom overlap
Functional constipation	Toilet routines, stool-withholding, school toilet avoidance, fiber/fluid intake, psychological stress	Clinical guideline/reviews and systematic reviews of observational studies [18,19,23,48]	The systematic review [48] included 11 pediatric studies, and most reported a significant stress–constipation association; heterogeneity precluded a reliable pooled effect estimate.	Moderate clinical support for bowel/toilet routines, lower-certainty observational evidence for psychological stress
Functional nausea	Stress/anxiety, sleep disruption, interoceptive processes, eating regularity	Predominantly observational evidence [22,38,42,49]	The pediatric sleep systematic review [38] identified increased sleep disturbance in functional nausea and reported markedly greater sleep problems in DGBI patients with co-occurring nausea in one included study (OR 2.93, *p* < 0.001).	Low certainty, sparse diagnosis-specific evidence

**Table 2:** Evidence base for circadian regulation of gastrointestinal function.

Gastrointestinal Process	Main Evidence Source	Population/Model	Evidence Relevant to Pediatric DGBI
Central and peripheral gastrointestinal clocks	Experimental and mechanistic studies summarized in reviews	Mainly animal models and adults [25,26,28]	Indirect; direct pediatric evidence is limited
Gastrointestinal motility and secretion	Experimental and clinical evidence summarized in reviews	Mainly animal models and adults [25,26,27,28]	Limited; direct evidence in children with DGBI is scarce
Circadian regulation of enteroendocrine hormones	Experimental and mechanistic studies	Mainly animal models and adults [25,26]	Indirect; pediatric DGBI relevance remains uncertain
Intestinal epithelial barrier and permeability	Experimental studies and reviews	Predominantly animal models; limited adult human evidence [26,29]	Insufficient direct pediatric evidence
Gut microbiota rhythmicity	Experimental and observational studies	Mainly animal models and adults; emerging pediatric evidence [26,30,31]	Emerging but insufficient for pediatric DGBI
Social jetlag and gastrointestinal outcomes	Observational evidence	Pediatric/adolescent evidence is mainly related to sleep, dietary, and metabolic outcomes; gastrointestinal evidence is limited [25,29]	Mainly indirect; direct pediatric DGBI evidence is scarce
Circadian disruption and DGBI	Mechanistic and clinical evidence	Mainly adults and animal models [25,26,27,28,29,30]	Insufficient direct pediatric evidence
Gut microbiota in children with sleep-related disorders	Systematic review	Pediatric population [31]	Limited pediatric evidence; direct DGBI outcomes remain insufficiently characterized

**Table 3:** Major confounding factors affecting the assessment of circadian variation in the pediatric gut microbiome [26,27,28,29,30,31].

Potential Confounder	Potential Influence on Gut Microbiota	Relevance to Circadian/Microbiome Studies
Sampling time	Microbial abundance, composition, and metabolite production may vary throughout the day	**High**; sampling at different times may be misinterpreted as inter-individual differences
Antibiotic exposure	May substantially alter microbial composition, diversity, and metabolic activity	**High**; recent or ongoing antibiotic use should be documented and considered in analyses
Age	The gut microbiome undergoes substantial developmental changes during childhood and adolescence	**High**; age-matched or age-adjusted comparisons are important
Pubertal stage	Hormonal and metabolic changes during puberty may influence microbial composition and host–microbiota interactions	**Important**, particularly in adolescent populations
Diet	Dietary composition, fiber intake, macronutrient distribution, food processing, and meal timing influence microbial composition and metabolites	**High**; dietary patterns may independently affect both circadian rhythms and the microbiome
Meal timing	Feeding schedules can directly influence microbial rhythmicity and metabolic activity	**High** in studies investigating circadian regulation
Sleep/circadian pattern	Sleep duration, sleep timing, and social jetlag may be associated with microbial changes	**Primary exposure of interest**, but difficult to distinguish from related behavioral factors
Medications and other exposures	Various medications and lifestyle factors may modify gut microbial composition	**Important**; medication use and relevant co-exposures should be reported

**Table 4:** Evidence base for dietary factors and circadian mechanisms and interventions in pediatric disorders of gut–brain interaction.

Gastrointestinal Process	Main Evidence Source	Population/Model	Evidence Relevant to Pediatric DGBI
Central and peripheral gastrointestinal clocks	Experimental and mechanistic studies summarized in reviews	Mainly animal models and adults [25,26,28]	**Indirect; direct pediatric evidence is limited**
Gastrointestinal motility and secretion	Experimental and clinical evidence summarized in reviews	Mainly animal models and adults [25,26,27,28]	**Limited; direct evidence in children with DGBI is scarce**
Circadian regulation of enteroendocrine hormones	Experimental and mechanistic studies	Mainly animal models and adults [25,26]	**Indirect; pediatric DGBI relevance remains uncertain**
Intestinal epithelial barrier and permeability	Experimental studies and reviews	Predominantly animal models; limited adult human evidence [26,29]	**Insufficient direct pediatric evidence**
Gut microbiota rhythmicity	Experimental and observational studies	Mainly animal models and adults; emerging pediatric evidence [26,30,31]	**Emerging but insufficient for pediatric DGBI**
Social jetlag and gastrointestinal outcomes	Observational evidence	Pediatric/adolescent evidence is mainly related to sleep, dietary, and metabolic outcomes; gastrointestinal evidence is limited [25,29]	**Very limited direct evidence for DGBI**
Circadian disruption and DGBI	Mechanistic and clinical evidence	Mainly adults and animal models [25,26,27,28,29,30]	**Insufficient direct pediatric evidence**
Gut microbiota in children with sleep-related disorders	Systematic review	Pediatric population [31]	**Limited pediatric evidence; direct DGBI outcomes remain insufficiently characterized**

**Table 5:** Reciprocal interactions between sleep, stress, circadian rhythm, and diet in pediatric DGBIs [34,35,36,37].

Interaction	Potential Pathway	Potential Relevance to Pediatric DGBIs	Level of Evidence in Pediatric DGBIs
**Stress** ↔ **Sleep**	HPA-axis activation, sympathetic activation, sleep fragmentation, altered stress reactivity	May contribute to visceral hypersensitivity, altered gastrointestinal motility, and symptom amplification	**Moderate**
**Stress** → **Gastrointestinal function**	HPA-axis and autonomic activation, immune signaling, altered intestinal barrier function	May contribute to abdominal pain and altered bowel habits in susceptible children	**Moderate**
**Sleep** → **Stress reactivity**	Increased HPA-axis and autonomic reactivity following insufficient or disrupted sleep	May reinforce psychological and gastrointestinal symptom burden	**Low-Moderate**
**Circadian rhythm** ↔ **Meal timing**	Peripheral clock regulation, timing of food intake, metabolic and gastrointestinal signaling	Irregular meals, breakfast skipping, and delayed eating may contribute to circadian misalignment; direct links with pediatric DGBIs remain uncertain	**Low**
**Circadian rhythm** ↔ **Sleep**	Irregular sleep–wake schedules, social jetlag, altered peripheral clock synchronization	May indirectly influence gastrointestinal symptoms through sleep and behavioral pathways	**Low**
**Diet** ↔ **Sleep**	Dietary composition, meal timing, appetite regulation, food preferences	Sleep disruption may promote less favorable dietary patterns, while dietary habits may influence sleep quality	**Low-Moderate**
**Diet** → **Gut microbiota**	Substrate availability, fermentation, SCFA production, microbial metabolism	Dietary patterns may influence microbial function relevant to gut–brain signaling and gastrointestinal symptoms	**Moderate**
**Sleep/Stress/Circadian rhythm** → **Gut microbiota**	Altered microbial composition and function, microbial metabolites, immune and metabolic signaling	Potential pathway linking lifestyle disruption with DGBI symptoms, but pediatric evidence is heterogeneous	**Low**
**Multiple factors** → **Gut–brain signaling**	Convergent neuroendocrine, autonomic, immune, metabolic, and microbial pathways	Sleep, stress, circadian timing, and diet may reinforce one another and contribute to symptom persistence	**Low**
**Integrated lifestyle factors** → **DGBI symptoms**	Reciprocal interactions among behavioral, neuroendocrine, autonomic, and microbial pathways	Supports a holistic clinical approach, but evidence for superiority of multicomponent interventions is currently insufficient	

## Data Availability

No new data were created or analyzed in this study. Data sharing is not applicable to this article.
